# Regional geographies and public health lessons of the COVID-19 pandemic in the Arctic

**DOI:** 10.3389/fpubh.2023.1324105

**Published:** 2024-01-08

**Authors:** Sweta Tiwari, Andrey N. Petrov, Nikolay Golosov, Michele Devlin, Mark Welford, John DeGroote, Tatiana Degai, Stanislav Ksenofontov

**Affiliations:** ^1^ARCTICenter, University of Northern Iowa, Cedar Falls, IA, United States; ^2^Department of Geography, University of Northern Iowa, Cedar Falls, IA, United States; ^3^Department of Geography, Pennsylvania State University, University Park, PA, United States; ^4^United States Army War College, Carlisle, PA, United States; ^5^Department of Anthropology, University of Victoria, Victoria, BC, Canada

**Keywords:** Arctic, COVID-19 pandemic, vulnerability, public health, indigenous, vaccination

## Abstract

**Objectives:**

This study examines the COVID-19 pandemic’s spatiotemporal dynamics in 52 sub-regions in eight Arctic states. This study further investigates the potential impact of early vaccination coverage on subsequent COVID-19 outcomes within these regions, potentially revealing public health insights of global significance.

**Methods:**

We assessed the outcomes of the COVID-19 pandemic in Arctic sub-regions using three key epidemiological variables: confirmed cases, confirmed deaths, and case fatality ratio (CFR), along with vaccination rates to evaluate the effectiveness of the early vaccination campaign on the later dynamics of COVID-19 outcomes in these regions.

**Results:**

From February 2020 to February 2023, the Arctic experienced five distinct waves of COVID-19 infections and fatalities. However, most Arctic regions consistently maintained Case Fatality Ratios (CFRs) below their respective national levels throughout these waves. Further, the regression analysis indicated that the impact of initial vaccination coverage on subsequent cumulative mortality rates and Case Fatality Ratio (CFR) was inverse and statistically significant. A common trend was the delayed onset of the pandemic in the Arctic due to its remoteness. A few regions, including Greenland, Iceland, the Faroe Islands, Northern Canada, Finland, and Norway, experienced isolated spikes in cases at the beginning of the pandemic with minimal or no fatalities. In contrast, Alaska, Northern Sweden, and Russia had generally high death rates, with surges in cases and fatalities.

**Conclusion:**

Analyzing COVID-19 data from 52 Arctic subregions shows significant spatial and temporal variations in the pandemic’s severity. Greenland, Iceland, the Faroe Islands, Northern Canada, Finland, and Norway exemplify successful pandemic management models characterized by low cases and deaths. These outcomes can be attributed to successful vaccination campaigns, and proactive public health initiatives along the delayed onset of the pandemic, which reduced the impact of COVID-19, given structural and population vulnerabilities. Thus, the Arctic experience of COVID-19 informs preparedness for future pandemic-like public health emergencies in remote regions and marginalized communities worldwide that share similar contexts.

## 1 Introduction

In late December 2019, a cluster of pneumonia-like cases was reported in Wuhan, the capital of Hubei Province, China (1, 2). The etiological agent in those cases was a novel coronavirus, known as SARS-COV-2, a group of RNA viruses that causes mild to severe respiratory infections in humans (1, 2). Despite the effort to contain the local outbreak in Wuhan, the virus spread quickly in other parts of mainland China and then the rest of the world, infecting more than 118,000 individuals in 114 countries and killing over 4,200 people just in the first 2 months of the outbreak (3, 4). The exponential spread of acute respiratory disease (popularly known as COVID-19) due to the SARS-COV-2 virus and the disease’s wider geographic diffusion led the WHO to declare COVID-19 a global pandemic on March 11, 2020 (2, 4). Over time the virus has mutated into many variants. Among them, WHO designated the Alpha, Beta, Gamma, Delta, and Omicron parent lineage as variants of concern based on their high transmissibility and virulent nature that can undermine the effectiveness of public health and social measures, including vaccines and therapeutics (5). Because of these variants, the whole world, including remote regions like the Arctic, experienced numerous epidemiological waves of infections and deaths (6–8). As of June 21, 2023, more than 768 million cases and 6.9 million deaths, globally, have been confirmed due to the pandemic (9). Whereas total excess deaths (defined as the difference between the observed numbers of deaths in specific time periods and expected numbers of deaths in the same time period) associated with COVID-19 for 2020 and 2021 was approximately 14.9 million, with 84% of those excess deaths occurring in the Americas, Europe and Southeast Asia (10, 11).

The first confirmed COVID-19 case in the Arctic was reported in February 2020 (12). To control the initial outbreak from spreading rapidly, most Arctic countries (excluding Russia and Sweden), introduced and strictly imposed COVID-19 public health containment measures during the first year of the pandemic (12–14). As the pandemic progressed and restrictions loosened, the Arctic also endured a significant burden of morbidity and mortality (8). As compared to the first year, in the second year of the pandemic, the Arctic reported a 205.8 and 334.8 percent increase in confirmed cases and deaths, respectively, (8). The Arctic COVID-19 epidemiologic curve shows at least four distinct waves identified as the first, second, Delta, and Omicron waves by Petrov et al. (15) resulting in over 2 million confirmed cases and approximately 28,000 deaths (16). Each wave’s temporal trend, magnitude, and severeness differed noticeably across the Arctic regions, while later waves were more aggressive; both Delta and Omicron parent lineage was more contagious and took more life in the Arctic than earlier strains of the virus (8, 15).

Drawing from historical experiences with previous pandemics (such as the 1918 flu, smallpox, tuberculosis, and the 2009 H1N1 flu), it becomes evident that Arctic residents are highly vulnerable to adverse COVID-19 outcomes (13, 17–20). Epidemiologically speaking, a wide array of determinants escalates the risk of severe COVID-19 infection and elevated mortality rates in the Arctic (21, 22). These include insufficient civic infrastructure (e.g., transportation, housing, sewage systems, healthcare facilities, etc.,), resource-dependent economies and healthcare systems, geographical barriers, the lingering legacy of colonialism, and a decade of marginalization (19, 22–24). Due to these vulnerabilities, Arctic residents, particularly the Indigenous population, shoulder an inequitable burden of chronic health conditions, such as diabetes, cardiovascular disease, and respiratory illnesses which have further amplified their susceptibility to severe COVID-19 health consequences (19, 21, 22, 25).

To date, however, it has been documented that even though vulnerable, Arctic communities (e.g., Alaska, Northern Canada, and Greenland) have curbed the expected dire epidemiological impacts (8, 15). By employing their ancestral knowledge, collective wisdom, and lessons from past pandemic experiences (21, 26, 27), the Arctic communities, especially Indigenous communities, have implemented proactive initiatives (such as community-wide lockdowns, stringent travel protocols, and rigorous adherence to COVID-19 guidelines) to limit the outbreak of the virus and to protect their vulnerable members (24, 28, 29). These initiatives have been found to be coupled with extensive awareness and vaccination campaigns, involving collaboration among different stakeholders like government officials, community leaders, NonGovernmental organizations (NGOs), and the general public (26, 30–33). Additionally, traditional healing practices, herbal medicines, and culturally appropriate interventions were found to be incorporated into Indigenous healthcare (24, 34, 35). Indigenous communities’ proactive leadership, grounded in the principle of self-determination in addition to customary practices, and Indigenous knowledge systems not only saved many lives but also highlighted the necessity and importance of healthcare approaches that are culturally attuned and responsive (20, 28, 36, 37).

The distinct combination of remoteness, vulnerable populations, and the Arctic communities’ resilience has rendered it a significant focal point in COVID-19 research (20, 37, 38). This focus aids in comprehending pandemic dynamics and pinpointing effective response strategies. Consequently, it informs preparedness for future pandemic-like public health emergencies, both within Arctic communities and other remote regions sharing similar contexts globally. Acknowledging this, this study aims to comprehensively examine the spatiotemporal epidemiological dynamics of the COVID-19 pandemic across 52 Arctic sub-regions, spanning the timeframe from February 2020 to February 2023. Another objective of this research is to elucidate public health lessons, most particularly the potential influence of vaccination coverage during the initial phases of the pandemic on the subsequent trajectories of COVID-19 outcomes within the specific delineated subregions. This holds particular significance as the swift deployment of vaccines and mass vaccination have proven crucial in mitigating the potential repercussions of the COVID-19 pandemic in some Arctic jurisdictions, such as Alaska, Northern Canada, and Greenland, given Arctic communities were among the first places in the world to experience large-scale vaccination efforts (20, 29, 39).

The analysis of the epidemiological dynamics of a pandemic provides important insights into its outbreaks, case distribution over time, and among diverse regions or populations, enabling us to draw inferences about its magnitude, severity, and geographic pattern. This epidemiological information, combined with vulnerability and resilience assessments, is pivotal in devising effective containment and preventive health strategies (20). This further aids in anticipating healthcare requirements based on characteristics of vulnerable populations and long-term disease complications, as well as in resource allocation and the implementation of interventions as needed.

Numerous researchers have acknowledged the impact of the pandemic on Arctic communities (12, 15, 20, 40), including (29, 41, 42); nevertheless, none of their studies have comprehensively explored the epidemiological data of COVID-19 through the full three years of the pandemic. Petrov et al. (12, 15, 40) conducted an analysis of three COVID-19 waves and their outcomes across the eight aggregated Arctic regions. Their findings indicated that COVID-19 infections and mortality in these regions remained lower than at respective national levels. Tiwari et al. (20) also assessed the COVID-19 epidemiological outcomes concerning Alaska within the framework of pandemic vulnerability and resilience and showed that communities with greater resilience exhibit lower cumulative death rates per 100,000 individuals and a decreased case-fatality ratio. Similarly, Noahsen et al. (29) assessed the influence of a rigorous COVID-19 public health strategy in Greenland, implemented until risk groups were immunized. Their study found that non-pharmaceutical interventions (NPIs) effectively curtailed the widespread transmission of the SARS-CoV-2 virus, resulting in low COVID-19 mortality rates. In contrast, a study conducted by Krieger et al. (42) in Arkhangelsk, Northwest Russia, detected no connection between adhering to NPIs and contracting the virus during the pandemic’s initial year. Furthermore, Barik et al. (41) scrutinized the COVID-19 situation in the Arctic and Subarctic regions.

Though these studies have great significance, they have a few limitations. For instance, Noahsen et al. (29) and Krieger et al. (42) focused solely on the initial year of the pandemic (2020 to 2021). The COVID-19 health outcomes data employed in the Barik et al. (41) study is representative of the national level and lack differentiation between the Arctic and Subarctic levels. Similarly, Petrov and his colleagues’ studies of the COVID dynamics across the aggregated Arctic regions did not capture the differences at the sub-regional level (8, 12). In response to these constraints, this study advances upon existing Arctic COVID-19 public health research by investigating the temporal dynamics of COVID-19 outcomes within a more refined spatial context, encompassing 52 sub-regions. Furthermore, we broaden our analysis to encompass COVID-19 vaccination dynamics and its influence on the outcomes of the pandemic.

## 2 Data and methods

### 2.1 Spatial coverage

**Figure 1:**
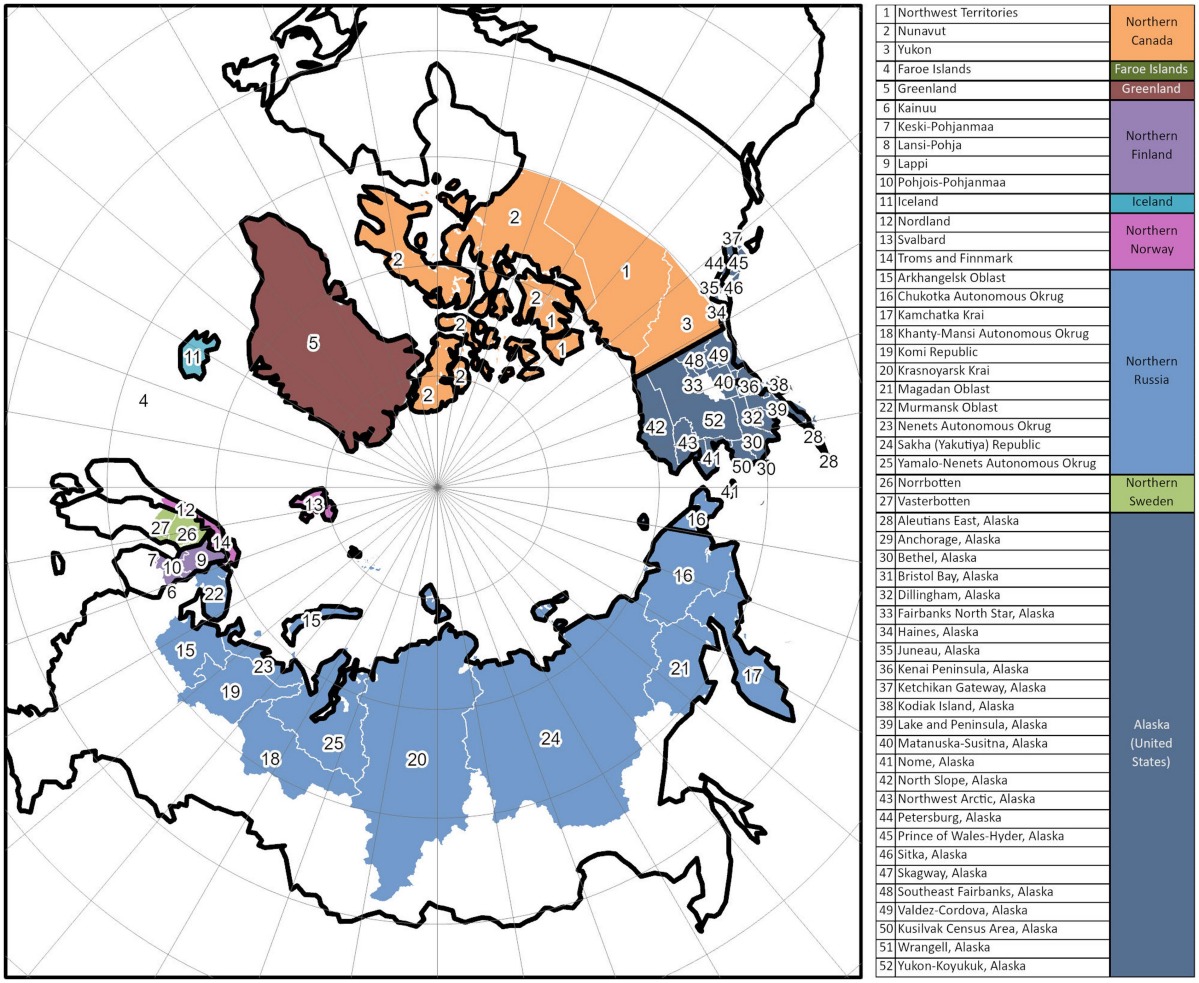
Study area.

For this study, spatial units of analysis encompass eight Arctic countries and 52 sub-regions of eight Arctic countries including Canada, the Kingdom of Denmark (Greenland and Faroe Islands), Finland, Iceland, Norway, Sweden, Russia, and the USA. The geographical boundaries of this study region (see Figure 1) closely follow the Arctic boundary established by the Arctic Human Development Report (43) and redefined by Jungsberg et al. (44).

### 2.2 Data sources

We developed a web-based information system called the Arctic Covid tracker (16) that automatically collected and disseminated information regarding COVID-19 epidemiological outcomes in the Arctic regions from various reliable sources. The Coronavirus Resource Center at Johns Hopkins University^1^ was the data source for Northern Canada, Greenland, Faroe Islands, Iceland, and the United States (Alaska), while the Public Health Agency of Sweden^2^ was for Northern Sweden, the Finnish Institute for Health and Welfare^3^ was for Northern Finland, the Government of the Russian Federation^4^ was for Russian Arctic and Verdens Gang^5^ was for Northern Norway. The COVID-19 data were extracted at 17:00 GMT daily from each mentioned source, then stored in a database and published daily via the Arctic COVID-19 dashboard.^6^

The collected database represents the best available data for the Arctic. Although different jurisdictions may have differences in data collection and reporting strategies, which could introduce bias in the results, the quality of the collected data at both national and regional levels (with a possible exception for Russia) adheres to the standards commonly used in Europe and North America (45, 46). Some sources, such as the Coronavirus Resource Center at Johns Hopkins University, have implemented additional quality control measures (47).

The database’s temporal coverage stretches from February 21, 2020, when the first COVID-19 case was reported in the Arctic, to February 2023, although the quality of data declined after summer 2022 due to inconsistency or halt in reporting in some jurisdictions.

Similarly, another web-based system called the ArcticVax tracker (48) was developed to collect and communicate COVID-19 vaccination information for 42 Arctic sub-regions. The vaccination data for Sweden’s Arctic regions (i.e., Västerbotten & Norrbotten) were collected from the Public Health Agency of Sweden. For other Nordic Arctic regions, finer-scale vaccination data were either unavailable or reported in different metrics. In addition, Finland reported the COVID-19 deaths in different spatial units. Therefore, our statistical analysis was confined to the subset of 44 regions that have complete COVID-19 outcomes and vaccination data.

### 2.3 Method and variable definitions

In this study, the COVID-19 pandemic’s spatiotemporal dynamics and health consequences are assessed using three key epidemiological variables: confirmed cases, confirmed deaths, and case fatality ratio (CFR) (49). *Confirmed COVID-19 cases* are individuals, whether symptomatic or asymptomatic, detected with the SARS-CoV-2 virus in their clinical specimen (50). *Confirmed COVID-19 deaths* are the count of fatalities resulting from a clinical illness due to the SARS-CoV-2 virus (50). *Case Fatality Ratio*, in this study context, is the proportion of individuals dying from COVID-19 among all those diagnosed with the disease within the given time frame. To explore and compare trends in the COVID-19 outcomes, either cases or death or CFR, across the Arctic sub-regions, we analyzed their cumulative and 7-day moving average rates (i.e., rates equivalent to per 100,000 population for cases and deaths, and per 100 for CFR) over the designated period.

The vaccination trends for COVID-19 within Arctic regions were examined based on the percentage of fully vaccinated individuals. The definitions of “fully vaccinated” may vary across different Arctic jurisdictions. Nevertheless, in most of these regions, the term “fully vaccinated” typically denotes individuals who have received at least two doses of an mRNA vaccine (such as Moderna and Pfizer), or one dose of the Johnson & Johnson vaccine, or equivalent vaccinations to attain full protection against severe clinical illness or death caused by COVID-19 infections (51). Owing to the declining efficacy of vaccines against emerging COVID-19 strains (like the Delta variant) (51, 52), several jurisdictions now stipulate additional doses to fulfill the criteria for being “fully vaccinated.” However, it’s worth noting that the data employed in this study may not encompass these recent recommendations.

Some Arctic regions became among the first parts of the world to administer mass vaccination as early as December 2020. This effort, along with other factors, has been considered instrumental in weakening the impacts of the pandemic in remote Arctic communities (37). To examine the effectiveness of the early vaccination campaign on the later dynamics of COVID-19 outcomes in these subregions, we conducted simple correlation and regression analysis. We assessed dependent variables, including CFR and cumulative deaths per 100,000, for specified periods, i.e., January 2021–July 2021 and January 2021–July 2022, using the percentage of fully vaccinated individuals in January 2021–July 2021 as a predictor for these outcomes.

## 3 Results

### 3.1 Overall pandemic outcomes

The examination of key pandemic variables reveals a significant and varied impact of the pandemic across the Arctic regions in terms of morbidity and mortality (Table 1).

**Table 1:** Key COVID-19 pandemic outcomes by Arctic region and country (as of February 28, 2023).

Country/Territory	Cases (cumulative)	Deaths (cumulative)	Cases (per 100, 000)	Deaths (per 100,000)	CFR (%)
Arctic	2,713,063	29,664	22,183.8	242.6	1.1
Iceland	208,999	213	57,3,962	57.9	0.1
Greenland	11,971	21	21,367.2	37.5	0.2
Faroe Islands	34,658	28	71,464.2	57.7	0.1
*Denmark*	3,403,360	8,265	58,757.6	142.7	0.2
Alaska (USA)	306,617	1,486	43,266.6	209.7	0.5
*USA*	103,443,455	1,119,917	31,251.5	338.3	1.1
Northern Finland	156,468	n/a	19,683.5	n/a	n/aa
*Finland*	1,462,169	8,892	26,389.5	160.5	0.6
Northern Canada	20,031	61	14,513.7	44.2	0.3
*Canada*	4,602,806	51,405	12,195.4	136.2	1.1
Northern Norway	91,421	113	18,643.0	23.0	0.1
*Norway*	1,479,032	5,175	27,223.1	95.3	0.3
Northern Sweden	114,559	1,018	27,591.3	245.2	0.9
*Sweden*	2,697,827	23,662	26,713.1	234.3	0.9
Northern Russia	1,768,339	26,726	19,191.7	290.1	1.5
*Russia*	21,960,719	388,126	15,048.3	266.0	1.8

*Data for Denmark proper. **Finland reports fatalities using different spatial units than cases.

As of February 28, 2023, the Arctic experienced 22,183.8 positive cases and 242.6 fatalities per 100,000 population. Several regions, including Iceland, the Faroe Islands, and Alaska demonstrated cumulative case numbers higher than the Arctic average mostly due to the spread of various COVID variants, particularly Delta and Omicron later in the pandemic, along with the relaxation of preventive measures (such as travel protocols, contact tracing, mask mandates, and social distancing, among others). Northern regions of Canada, Norway, and Russia reported comparatively lower confirmed cases during the 3 years of the pandemic, although the underlying reasons may differ, ranging from lower infection rates to potential underreporting. The most elevated mortality rates were observed in Northern Russia (290.1 per 100,000), Northern Sweden (245.2), and Alaska (209.7). High mortality rates are also correlated with elevated Case Fatality Ratios (CFR) in these regions. While the CFR for the Arctic as a whole stood at 1.1%, it was 1.5% in Northern Russia, 0.9% in Northern Sweden, and 0.5% in Alaska. Higher CFR would be expected in the Arctic due to limited accessibility to healthcare facilities, the presence of vulnerable populations (such as individuals with preexisting health conditions), and potential difficulties or inconsistencies in implementing effective containment and healthcare measures. Notably, however, across all Arctic regions, the CFR remained below the national levels of their respective countries, an important fact that has been highlighted in the literature as a sign of resilience to the pandemic (8, 15, 37).

### 3.2 Five waves of COVID-19 in the Arctic

**Figure 2:**
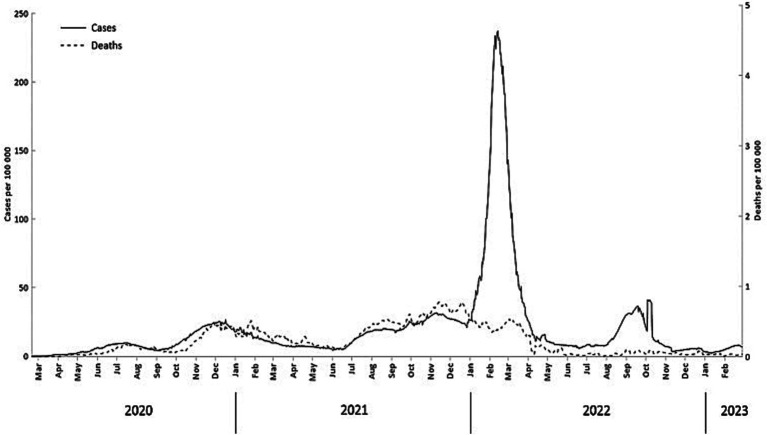
Daily confirmed COVID-19 cases and deaths (7-day moving average) (February 20, 2020- February 28, 2023).

As shown in Figure 2, the pandemic progressed in the Arctic through multiple “waves,” marked by surges in infection and deaths followed by significant declines sustained over specific periods. Notably, the pandemic had a relatively delayed onset in numerous Arctic regions, with the initial wave becoming apparent only in the summer of 2020 (40). This lag could be attributed to the remote nature of Arctic areas and stringent preventative policies implemented in some jurisdictions (28, 29, 36, 53). During the fall of 2020, most Arctic regions experienced a second wave in which they encountered a peak in COVID-19 infections and deaths in mid-December 2020, followed by a decline in the early months of 2021. From July to December 2021, the third wave due to the Delta variant gained momentum (8), resulting in record-high fatalities across all Arctic jurisdictions that surpassed those observed in both preceding and subsequent waves. Following shortly was the fourth wave caused by the Omicron variant which outstripped previous infection rates in the Arctic. This specific wave led to significant outbreaks in regions like the Faroe Islands and Iceland, both of which had experienced fewer COVID-19 cases during the earlier waves. However, the Omicron wave did not entail a significant increase in COVID-19 mortality (Figure 2). The fourth wave receded by the summer of 2022. A new resurgence in positive cases (the fifth wave) occurred during the fall of 2022 as multiple regions eased COVID-19 health measures. The uptick in infections during the fall highlights the ongoing existence of the COVID-19 pandemic in the Arctic.

**Figure 3:**
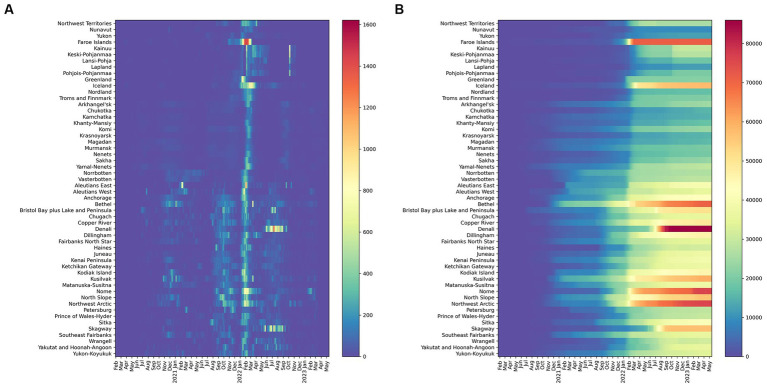
Confirmed daily COVID-19 cases per 100,000 **(A)** (left) 7-day moving average and **(B)** (Right) Cumulative Confirmed COVID-19 cases per 100,000 (February 20, 2020- February 28, 2023) Warmer colors correspond to more cases and cooler colors to fewer cases per 100,000.

**Figure 4:**
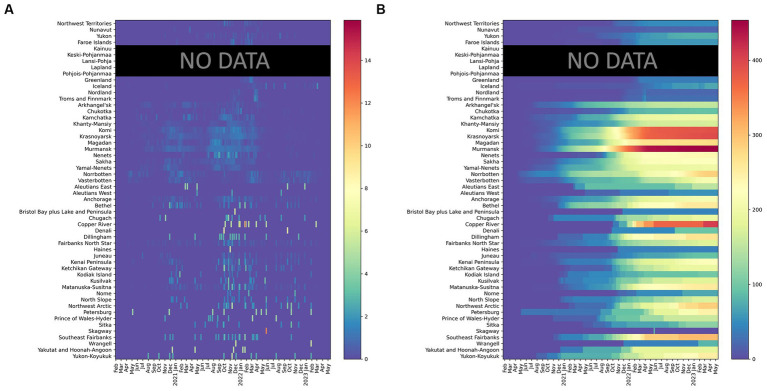
Confirmed COVID-19 deaths per 100,000 **(A)** (left) daily (7-day moving average) and **(B)** (right) cumulative in 52 regions (February 20, 2020- February 28, 2023). Finland aggregates fatalities by hospital districts, which differ from regions used for aggregating cases. Death rates for Finland are not reported.

**Figure 5:**
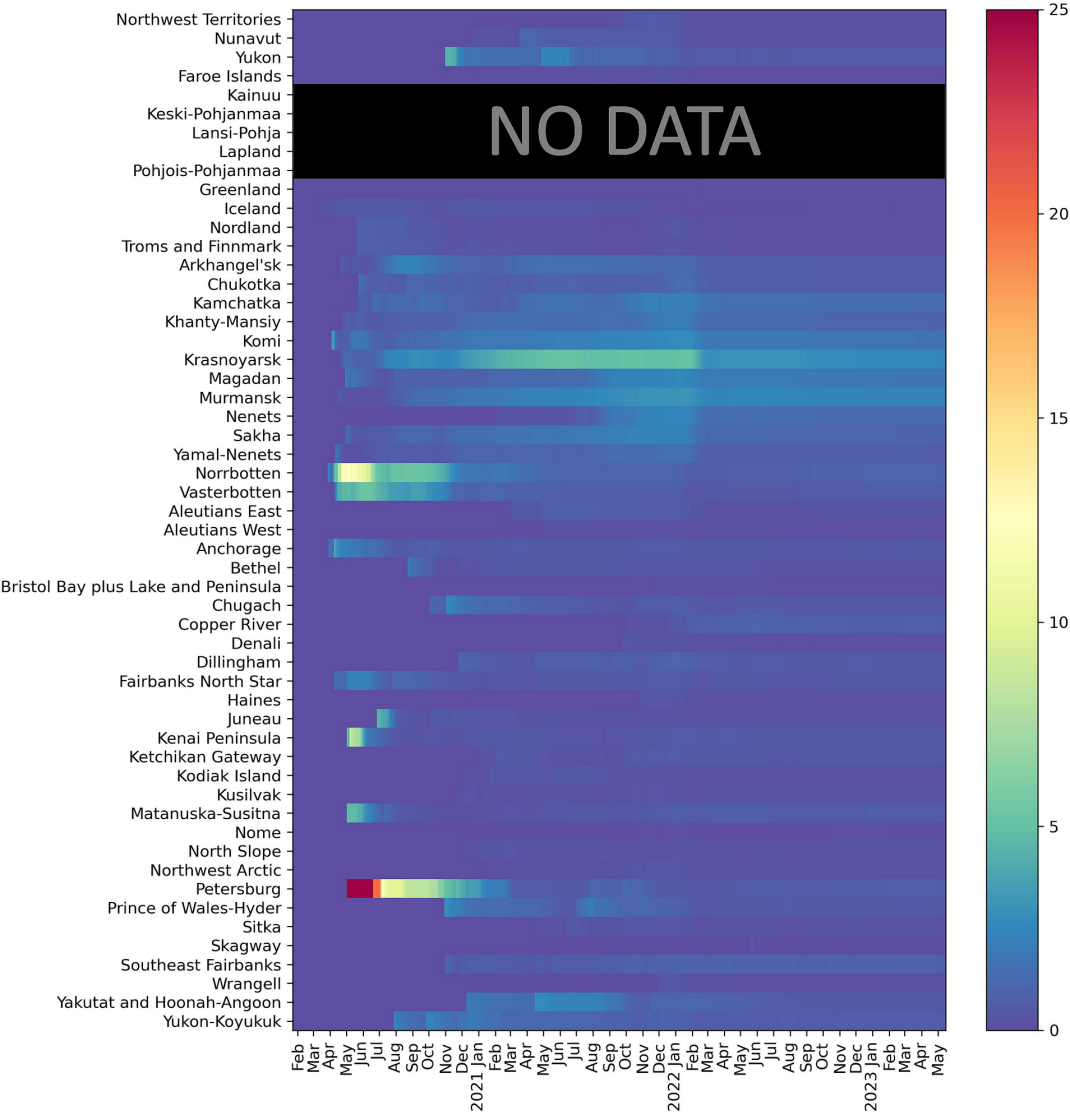
Case Fatality Ratio (February 20, 2020- February 28, 2023); Finland aggregates fatalities by hospital districts, which differ from regions used for aggregating cases. Death rates for Finland are not reported.

**Figure 6:**
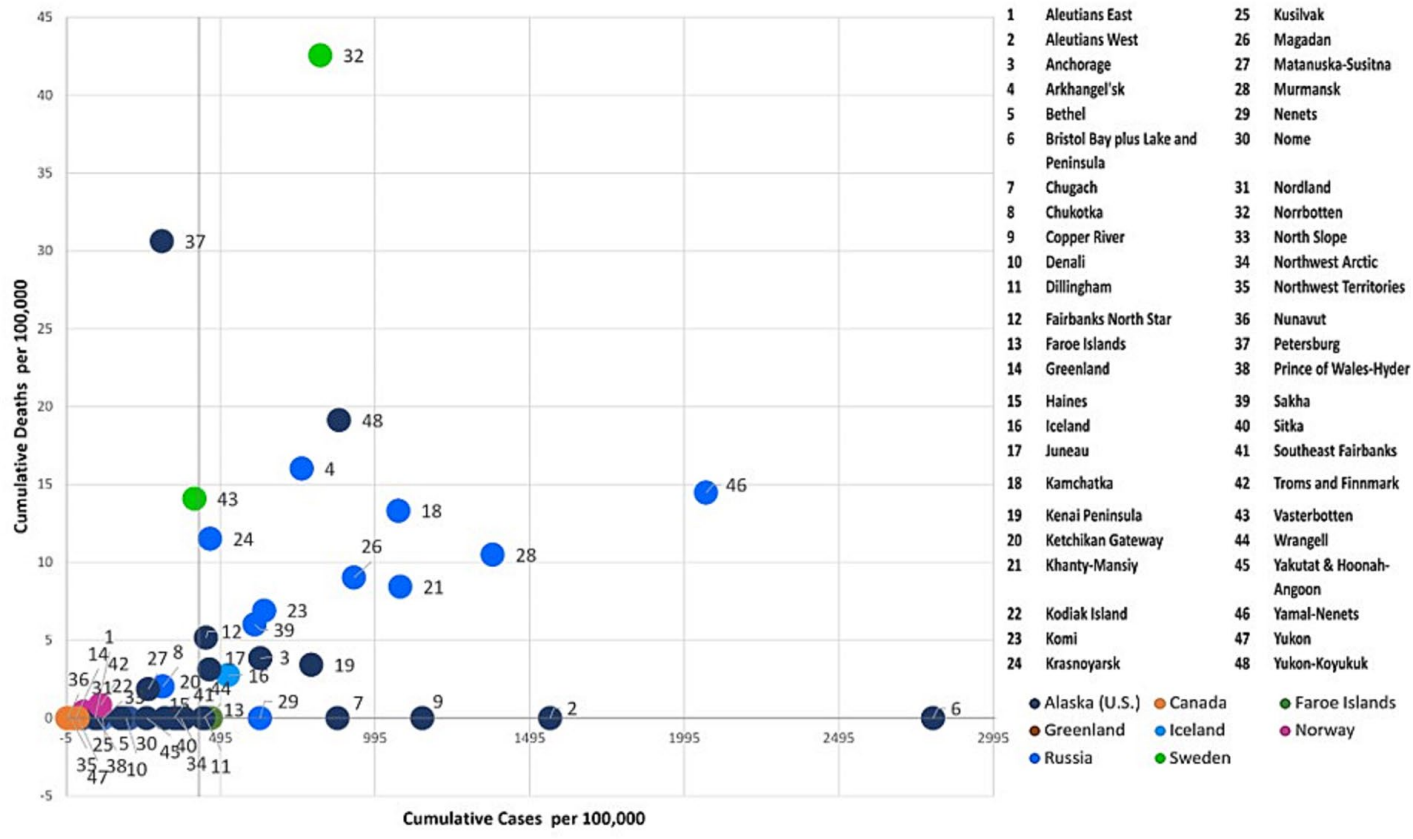
Regional typology of the COVID-19 outcomes as of July 31, 2020.

Figures 3–6 present a more disaggregated spatiotemporal view of the COVID-19 pandemic by plotting COVID-19 cases, deaths, and CFR across 52 regions and 36 months. The first graph (Figure 3A) depicts daily COVID-19 cases and is designed as a “heat map” with cooler colors corresponding to fewer cases per 100,000 and warmer colors indicating more case rates. The five waves are well identified in many or most Arctic regions, although considerable regional differences are also evident. Everywhere the onset of the pandemic was delayed (40). The earliest wave took place in Alaska and Sweden in the summer of 2020. The Delta and Omicron waves are very vivid, and the latter is observable in almost all regions. It is also characterized by the largest number of cases per 100,000. In Russia, this and other waves appeared to be slightly delayed (by about 2 weeks). The fifth, summer 2022 wave, has been substantial in Alaska, Finland, and Iceland, and, to a lesser extent, in Arctic Russia.

Figure 3B demonstrates the dynamic of cumulative COVID-19 cases. Although cases grew in all regions, the trend has been uneven both with respect to the start of a noticeable increase in recorded infections and in terms of subsequent rapid growth associated with COVID-19 waves. Notably, in some areas, the elevated levels of COVID-19 emerged earlier (e.g., parts of Alaska and Russia), while in others the start of the pandemic was much later (e.g., Nunavut, northern Finland, and Norway). The impacts of the Delta and Omicron waves are also evident, especially in regions where massive COVID-19 spikes took place in early 2022, such as the Faroe Islands.

Regional patterns of COVID-19 mortality are illustrated in Figure 4. Russian Arctic regions and some Alaska boroughs demonstrated the highest cumulative death rates per 100,000 (Figure 4B). Elevated rates early in the pandemic were observed in northern Sweden (Västerbotten and Norrbotten). The seven-day average death rate (Figure 4A) is more difficult to interpret, but it indicates a general rise in Russian regions during the pandemic waves, in particular, Delta and Omicron, as well as shows highly variable dynamics in Alaska subregions partially due to small population numbers.

Finally, CFR (Figure 5) in the Arctic has a typical pattern of high values right after the onset of the pandemic in a given region with subsequent subsidence as time elapsed - a picture typical for most regions of the world (54–56). The decline in CFR is especially significant during and after the Omicron wave. There is a well-noticeable spike in CFR in northern Sweden in the Spring–Summer of 2020 most likely attributable to relaxed anti-COVID-19 policies and lack of NPIs at the time (57). High CFRs are also seen across the Russian Arctic in 2021.

### 3.3 Regional typology of COVID-19 dynamics

**Figure 7:**
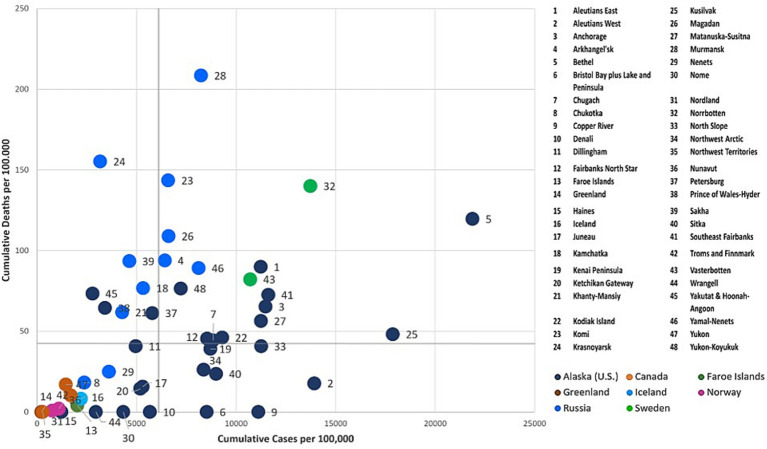
Regional typology of the COVID-19 outcomes as of July 31, 2021.

**Figure 8:**
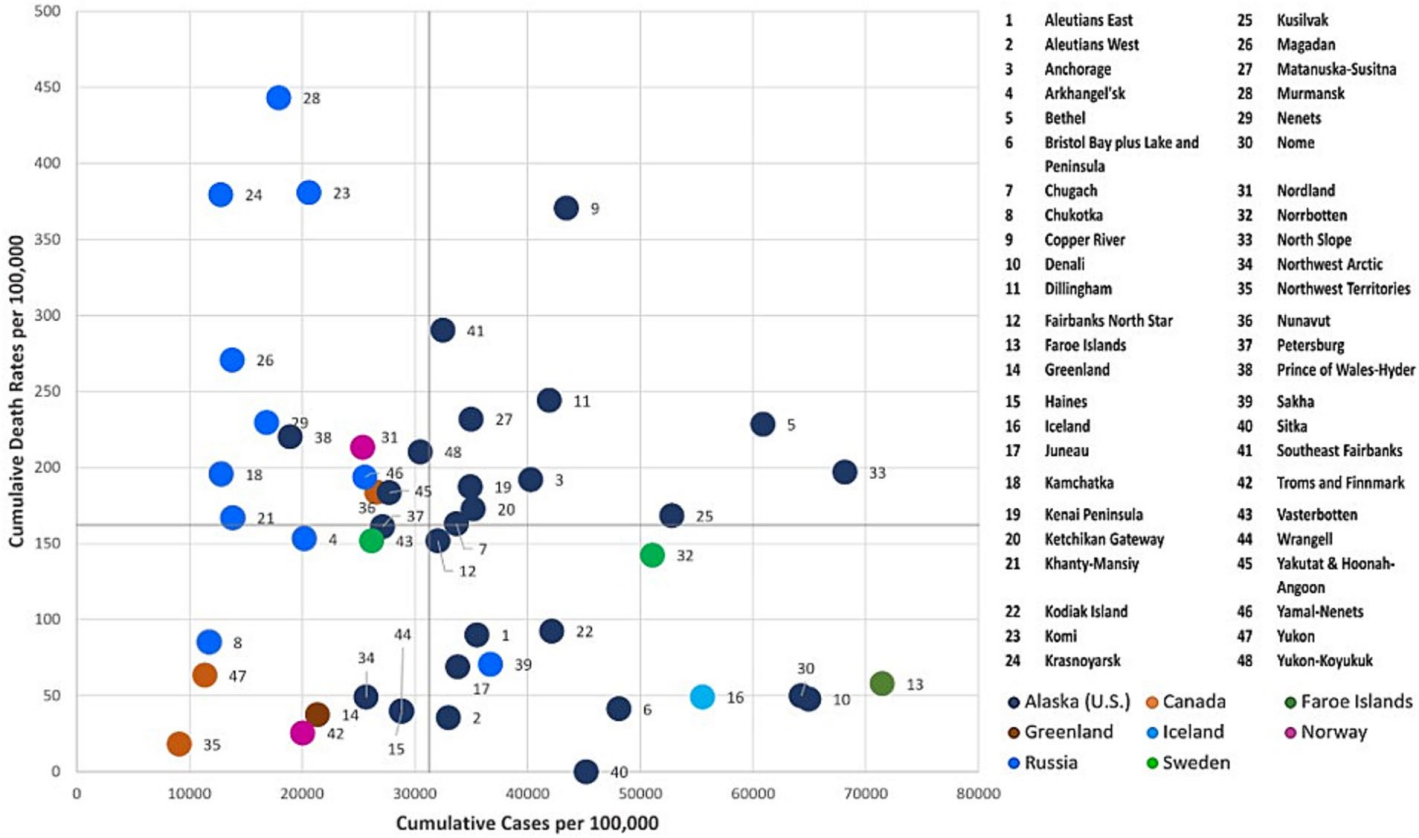
Regional typology of the COVID-19 outcomes as of July 31, 2022.

To further examine the dynamics of the COVID-19 pandemic over time, we combinedly assessed the two key indicators, cumulative COVID-19 cases and deaths per 100,000 across the Arctic at three given points in time (July 31, 2020, July 31, 2021, and July 31, 2022) using the four-quadrant typology (i.e., High-High, High-Low, Low-High, and Low-Low). Each Arctic region was classified into quadrants (Figures 6–8) using quantiles, effectively pinpointing high- and low-risk zones. This exploratory technique helps in comprehending the intricate interplay between the effectiveness of COVID-19 public health interventions and the differing degrees of COVID-19 outcomes observed among Arctic regions.

#### 3.3.1 Low cases-low deaths cluster

This quadrant characterizes regions with relatively low rates of COVID-19 cases and deaths. During the early stages of the pandemic (see Figure 6), most Arctic regions did not witness COVID-19-related infections and deaths. However, Iceland, the Faroe Islands, Northern Norway, and Northern Finland did experience higher early incidence. Still, stringent quarantines and other protective measures were effective in preserving lives, resulting in no reported deaths in these regions. Northern Russia, Northern Sweden, and Alaska all experienced prolonged initial waves of infections, which subsequently led to increased mortality rates largely attributed to the implementation of relatively lax or inconsistent public health measures (57, 58).

Greenland and Canadian Arctic jurisdictions, including Yukon, Northwest Territories, and Nunavut, reported relatively low rates of COVID-19 cases and few deaths over the course of all 3 years (Figures 6–8) of the pandemic. These regions effectively implemented preventive and containment strategies, such as isolation, quarantine, travel restrictions, and mass vaccination campaigns, to minimize the pandemic’s impact (28, 29, 36, 53). In addition, there was relatively little COVID-19 impact in Indigenous boroughs of Alaska, including the North Slope, Northwest Arctic, and Nome, during the pandemic’s initial year, with few cases and isolated deaths. However, after the summer of 2021 (Figures 7, 8), there was a significant increase in caseloads in these regions (except Northwest Arctic) with occasional spikes in fatalities.

#### 3.3.2 High cases -low deaths cluster

Cumulatively, this quadrant depicts regions with higher COVID-19 case rates and corresponding lower death rates. During the initial year of the pandemic (as of July 31st, 2020), Faroe Islands, Nenets, and numerous coastal Alaska regions such as Dilinham, Wrangell, Chugach, Copper River, Bristol Bay & Lake, and Peninsula, as well as Aleutians West, fell into this category (Figure 6). These regions encountered an early onset of the pandemic with escalating infections. Despite the high case numbers, the implemented COVID-19 containment measures, including stringent lockdowns and quarantines, have likely been effective in reducing the death toll. Even after the gradual easing of restrictions, Aleutians West and Bristol Bay, and Lake & Peninsula, have persisted as cold spots for deaths throughout the pandemic (i.e., in the years 2021 and 2022) (Figures 7, 8). This persistence may be attributable to their effective healthcare response, encompassing timely testing, meticulous contact tracing, and a successful vaccination campaign that has averted severe outcomes.

#### 3.3.3 Low cases - high deaths cluster

This quadrant characterizes regions with a relatively low number of reported COVID-19 cases per 100,000 but a relatively high number of fatalities. Over the progression of the pandemic, most regions of the Russian Arctic have fallen into this category (Figures 7, 8). Some of these regions, including Magadan, Kamchatka, Komi, Krasnoyarsk, Murmansk, Khanty-Mansiysk, and Yamal-Nenets, continued to experience higher fatalities, despite a decline in reported new cases after first year (Figures 7, 8). Elevated death rates in Northern Russia could stem from inconsistent quarantine measures, constrained healthcare capacities, and restricted availability and uptake of vaccines in remote areas. Similarly, a few Alaskan jurisdictions, such as Petersburg, Matanuska-Susitna, and Yakutat & Hoonah-Angoon, also saw higher mortality despite having a low to moderate infection rate over 3 years (Figures 6–8). In Northern Norway, regions like Nordland, and Troms and Finnmark witnessed a sudden rise in mortality rates during the initial year, which significantly decreased following the implementation of an aggressive prevention policy. Norway initially eased prevention measures in the summer of 2021, but later reinstated most of these measures (59), probably leading to lower cases and mortality rates per 100,000 after the summer of 2021 (Figure 7). In Northern Sweden, Västerbotten observed relatively lower-case rates at the outset of the pandemic, but high death rates until the summer of 2021. Following a robust second wave with a relatively high CFR, Sweden enacted COVID-19 measures and restrictions in January 2021 (60), likely resulting in reduced deaths in Västerbotten (unlike in Norrbotten) compared to the second wave. These lower rates persisted into the third year of the pandemic, accompanied by a decrease in cases per 100,000 (Figure 8).

#### 3.3.4 High cases -high deaths cluster

**Figure 9:**
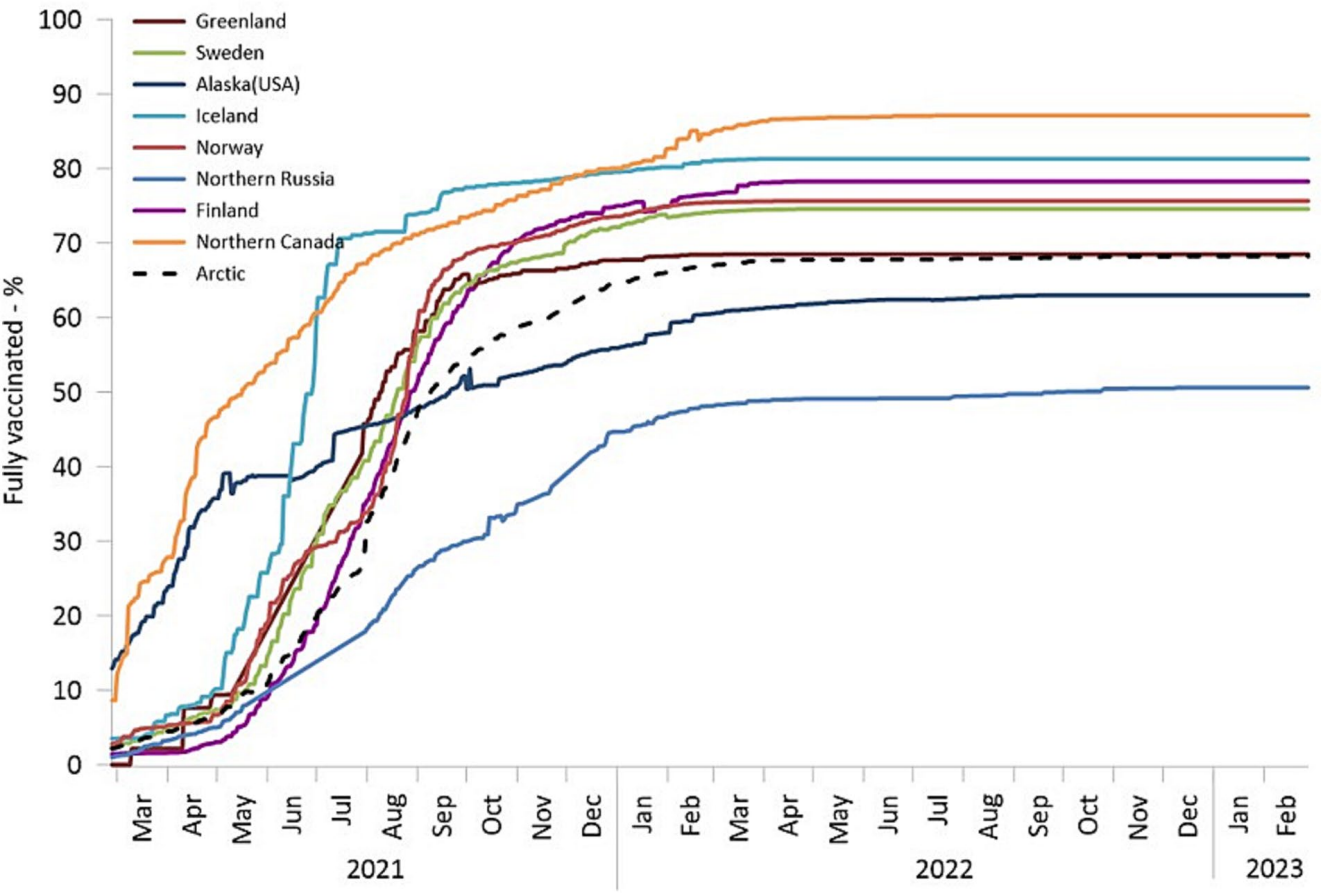
The percentage of fully vaccinated individuals among the total population. Finland, Norway, and Sweden are depicted using countrywide data.

Iceland, Norrbotten, many Alaska regions such as Fairbanks North Star, Juneau, Anchorage, and Yukon-Koyukuk, and all regions in the Russian Arctic (except for Nenets and Chukotka) initially reported a significant number of COVID-19 cases and fatalities (Figure 7). Many of these regions possess densely populated urban centers, leading to elevated transmission rates and severe outcomes. Norrbotten continued to experience relatively high death rates per 100,000 during the second and third years of the pandemic (Figures 8, 9). Iceland implemented stricter prevention measures in both the private and public spheres during the spring of 2021 (61), followed by variable prevention measures based on epidemiological trends and mass vaccination campaigns throughout the year, resulting in decreased death rates after 2021 (Figures 8, 9). Several southern Alaska regions, the Northwest Arctic and Southeast Fairbanks, reported elevated rates of infections and deaths during the second year and later in the pandemic (Figures 7, 8). These can be attributed to the relaxation of COVID-19 restrictions, varying enforcement of public health interventions, and slower growth in vaccine uptake (12).

### 3.4 Spatiotemporal dynamics of vaccination in the Arctic

As of September 2022, nearly 70% of Arctic residents had received full vaccination per the criteria set forth by their respective jurisdictions. However, the spatial and temporal patterns of COVID-19 vaccine uptake exhibited variations. Alaska and Northern Canada initiated their vaccination campaigns as early as December 2020. By May 2021, Northern Canada had achieved a vaccination rate of 50%. Similarly, by May, more than 60 percent of adults (i.e., aged 16 years and older) residing in certain Alaskan boroughs, including Aleutians East Borough, Skagway Municipality, Sitka City and Borough, and Yukon-Koyukuk Census Area, had received at least one vaccine dose (48). Notably, Alaska and northern Canada, represent cases of very early and massive vaccination efforts, often co-managed by the public and tribal health authorities (30, 37).

In Alaska, although vaccination rates were initially very robust and rapid, there was a swift drop off in uptake during the subsequent months. Conversely, there was a delay in vaccine rollout in other Arctic regions; however, these regions (with the exception of Northern Russia) promptly increased vaccination rates, achieving a coverage level of 60–70% by the end of 2021. Northern Russia’s vaccination campaign progressed slowly and faced limited success, partially due to increased vaccine hesitancy (62) and resistance (63). It could be argued that rapid adoption of vaccines can be part of a robust response to the pandemic in remote areas. Previously, we argued that the remoteness of Arctic regions carries both a “blessing” associated with a delayed onset of the pandemic, and a “curse” embedded in the higher vulnerability of remote places to the pandemic (27). In this context, an early mass vaccination campaign can be an effective solution to moderate the curse by vaccinating ahead of a major COVID-19 wave, thus lessening the overall pandemic impacts, especially mortality. Therefore, one can look for a relationship between early vaccination rates and pandemic outcomes in remote areas where vaccines were distributed in advance of other places.

### 3.5 Impact of early vaccination on pandemic outcomes

To investigate the potential impact of initial vaccination coverage on subsequent COVID-19 outcomes (cases, deaths, and CFR) within Arctic regions, we conducted a linear regression analysis. We analyzed only regions (*n* = 44) with complete data for all considered variables. Prior to conducting the regression, we performed Pearson correlation calculations to discern any possible associations between the percentage of fully vaccinated individuals and COVID-19 outcomes. Our analysis encompassed cumulative data for the percentage of full vaccination spanning the period from January 2021 to July 2021 (early vaccination period), as well as COVID-19 outcomes, (i.e., cumulative cases, and deaths per 100,000 and the CFR in percent), for two distinct temporal intervals: January 2021 to July 2021 (period concurrent with first 6 months of vaccinations) and January 2021 to July 2022 (i.e., 18 months after the start of mass vaccinations). During this latter period, characterized by the dominance of the Delta and Omicron variants, more than 2,042,163 new cases were recorded, representing a substantial 583.3% increase. Hence, when analyzing the correlation between vaccination rates and mortality at different times, another factor to consider is the waves were caused by different variants, each having its intrinsic mortality rate.

For both temporal intervals (Table 2), there was a statistically significant negative association between fully vaccinated individuals (%) and cumulative deaths per 100,000 and the CFR (%). Notably, these associations strengthened significantly over time (*r* = −0.68 and − 0.73). Regarding COVID-19 cases, the relationship between cumulative cases per 100,000 and the percentage of fully vaccinated individuals during the period, from January 2021 to July 2021, was found to be nonsignificant. However, over the period from January 2021 to July 2022, this relationship has been moderately positive. These results implied that despite the surge in infection rates, earlier vaccination coverage exhibited significant efficacy in mitigating severe subsequent COVID-19 outcomes, particularly mortality and CFR, in the Arctic.

**Table 2:** Pearson correlation coefficients.

COVID-19 outcomes	Fully vaccinated individuals (%) (Correlation coefficient)	
	January 2021 to July 2021	January 2021 to July 2022
Cumulative cases per 100,000	0.11	0.46***
Cumulative death per 100,000	−0.48***	−0.68***
CFR (%)	−0.55***	−0.73***

*, **, *** equal statistical significance at 5, 1 and 0.1 percent levels. Here, the total number of observations (*N* = 44).

The correlation results were further confirmed by the regression analysis (see Tables 3, 4). During the period from January 2021 to July 2021, the observed effect size of the vaccination rate on the cumulative death rate per 100,000 individuals appeared to be small in magnitude (almost none) yet statistically significant (Table 3). However, when we conducted a regression analysis involving the cumulative mortality rates spanning from January 2021 to July 2022 and initial vaccination coverage, the impact appeared notably stronger and statistically significant. In simpler terms, a mere 1 % rise in the fully vaccinated population led to a corresponding decrease in mortality by 3.10 per 100,000 individuals in the near future. Additionally, the initial vaccination coverage seemed to provide a more effective explanation for the variability observed in the cumulative death rate per 100,000 individuals during the latter period compared to the earlier one. This improvement was reflected in the Adjusted R-squared value, which increased from 21.6 percent to 45.3 percent.

**Table 3:** Simple linear regression analysis between COVID-19 vaccination and death rates.

		Dependent variable = Cumulative death per 100,000	
Time period	Predictors = Fully vaccinated population (%)	Estimates	CI
January 2021 to July 2021	(Intercept)	0.06***	0.04–0.09
	Regression coefficient	−0.00***	−0.00 – −0.00	R^2^ / R^2^ adjusted	0.234 / 0.216	
January 2021 to July 2022	(Intercept)	270.85***	220.54–321.15
	Regression coefficient	−3.10***	−4.14 – −2.07	R^2^ / R^2^ adjusted	0.465 / 0.453	

CI stands for confidence interval. *, **, *** equal statistical significance at 5, 1, and 0.1 percent levels (* *p* < 0.05, ** *p* < 0.01, *** *p* < 0.001). *N* = 44.

**Table 4:** Simple linear regression analysis between COVID-19 vaccination and CFR rates.

		Dependent variable = CFR	
Time period	Predictors = Fully vaccinated population (%)	Estimates	CI
January 2021 to July 2021	(Intercept)	2.84***	1.94–3.74	Regression coefficient	−0.04***	−0.06 – −0.02	R^2^ / R^2^ adjusted	0.300 / 0.283	
January 2021 to July 2022	(Intercept)	1.90***	1.51–2.29	Regression coefficient	−0.03***	−0.04 – −0.02	R^2^ / R^2^ adjusted	0.527 / 0.516	

CI stands for Confidence Interval. *, **, *** equal statistical significance at 5, 1, and 0.1 percent levels (* *p* < 0.05, ** *p* < 0.01, *** *p* < 0.001). *N* = 44.

A similar trend was observed for the CFR (Table 4). Earlier vaccination efforts appeared to result in a reduction of CFR during the later period. Specifically, CFR decreased by 0.03 percent with each 1 % increase in the fully vaccinated population. Furthermore, the adjusted R-squared value reached 51.6 percent, indicating that earlier vaccination accounted for nearly half of the variation observed in the later CFR.

## 4 Discussion and conclusion

### 4.1 Regional dynamics and ‘models’

Over 3 years starting from February 2020 the global community, including the Arctic, has felt the epidemiological impact of COVID-19 and its various variants. This study indicates that the Arctic has witnessed five distinct waves of infections and fatalities due to the outbreak of the SARS-COV2 virus and its mutated strains, with Alpha, Delta, and Omicron and its subvariant BA.5 (i.e., fifth wave) having higher prevalence during this timeframe. When comparing these strains, we found that the Delta wave was more severe, bringing more deaths in the Arctic that led to higher CFRs while Omicron resulted in the highest surge in positive cases, resulting in a steep rise in recorded infections but fewer deaths and declined CFR.

The examination of reported COVID-19 cases and fatalities from 52 Arctic subregions reveals that the pandemic’s severity exhibited substantial spatial and temporal variations. A common trend was the delayed start of the pandemic in the Arctic - a result of its remoteness. An ability and desire to hold off the pandemic’s offset have certainly given Arctic regions an advantage, despite their well-recognized vulnerability (21, 24, 26). A few regions managed the pandemic well: Greenland, Iceland, Faroe Islands, Northern Canada, Finland, and Norway witnessed isolated spikes of cases at the onset, which were swiftly contained with minimal or no fatalities. Most of these regions landed in the “low cases and low deaths” cluster of the regions - and represent the most successful model of dealing with the pandemic. Northern Russia, Northern Sweden, and Alaska showed different, but generally less successful models with more negative COVID-19 dynamics. Death rates in Northern Sweden and Northern Russia were generally high, with explosive surges in cases and death rates following the first and second waves. During the Delta Wave, the Arctic region saw the highest mortality rates, again with Northern Russia, Northern Sweden, and Alaska emerging as the leading regions in this regard. The Omicron variant and its subvariant waves resulted in substantial outbreaks in regions such as the Faroe Islands and Iceland.

### 4.2 Global and local public health lessons

Throughout the pandemic, however, mortality rates and CFR in most northern regions remained (apart from Russia) lower than those in the southern parts of their respective countries. In this respect, the Arctic’s pandemic response experience provides important lessons for informing public health interventions in remote regions across the globe. The combination of remoteness, proactive public health measures informed by prior pandemic experiences, and Indigenous knowledge enabled certain communities with high socioeconomic and health vulnerabilities to navigate the early stages of the pandemic effectively and be better prepared for the arrival of COVID-19 (24, 27, 39). Implementing early preventive measures that were culturally appropriate, such as placing the highest priority on protecting vulnerable elders from infectious disease, providing health education campaigns in native languages using tribally relevant imagery and themes, providing COVID-19 vaccination programs through fly-in/fly-out village nursing programs, and utilizing public outreach campaigns through popular local radio stations and social media sites were critically important in many of these Arctic Indigenous communities (8, 64, 65).

The remote geography of the Arctic and stringent preventive measures early in the pandemic delayed its onset in most of its regions (40), although it did not entirely avert the significant outbreaks of cases after the Fall of 2020 when public health and social measures were implemented inconsistently across the Arctic and globe (8, 12). Thereafter, most remote Arctic communities faced strenuous challenges in responding effectively to the rapidly dispersing pandemic due to constraints stemming from inadequate healthcare resources and limited infrastructures (8, 66).

Despite a rapid increase in infection rates mirroring those of their respective nations, most Arctic regions consistently maintained a low CFR, attributed possibly to the success of mass vaccination campaigns, as suggested by the analysis undertaken in the paper: regions with higher vaccination rates early in the pandemic tended to have lower mortality and CFR. Indeed, some remote Indigenous regions in Alaska and Northern Canada were among the first locales around the world where vaccines were widely distributed. Vaccination initiatives were widely embraced and adopted even in the face of a historical context marked by instances of coerced medical experimentation and abuse in these regions (8, 67).

The key lesson that the global public health community can learn from the Arctic Indigenous Peoples in the context of the COVID-19 pandemic is the significance of Indigenous self-determination in healthcare, community engagement, and Indigenous knowledge, which empowered these communities to establish their own strategies, campaigns, and priorities for addressing the crisis (24, 26, 33, 37, 64). Indigenous knowledge and the continuation of on-the-land practices, which encompass a wide range of traditional activities and customs, constitute an indispensable facet of Indigenous communities’ way of life, fostering their physical, mental, and spiritual well-being while also promoting cultural resilience and sustainability (32, 53). These underscore the significance of healthcare approaches that are culturally sensitive and adaptive which could potentially serve as a valuable instrument in post-COVID-19 rehabilitation and future pandemic preparedness (27, 28, 36, 37).

Learning from the Arctic may provide important insights for dealing with future pandemics in remote areas and Indigenous homelands. The relative geographic isolation of Arctic indigenous communities, which can be helpful in first delaying the arrival of infectious diseases into these communities, can sometimes create challenges later in receiving high-level treatment for these conditions in advanced cases. Thus, the Arctic’s relative success in addressing COVID fundamentally reinforces the urgency of enhancing remote-area public health services, improving access to medical care in underserved areas, bridging socioeconomic gaps, and closing Indigenous health disparities in the Arctic and around the world.

### 4.3 Limitations and future directions

There is no doubt that the epidemiological data and analysis presented in this study are pivotal in the realm of public health, providing essential guidance for disease surveillance, the formulation of preventive strategies, healthcare resource allocation, and rigorous research endeavors. This study, thus, aids policymakers, healthcare practitioners, and researchers with the knowledge required to make well-informed decisions aimed at enhancing the health and overall well-being of populations. However, the data and analyses conducted in this study have a few limitations. This study relied on publicly accessible datasets that could be susceptible to underreporting, misreporting, and inconsistencies. Though this could potentially introduce a degree of bias into the findings, the data integrity at both the national and regional levels conform to the standards typically employed in Europe and North America. Furthermore, in an effort to partially alleviate these data concerns, this paper computed cumulative rates and moving averages that reflect longer-term trends rather than short-term pandemic events. In our statistical analysis, we did not incorporate control variables, potentially leading to less efficient parameter estimates. Consequently, this implies that there may be some degree of uncertainty associated with our estimated parameters. Finally, the data analyzed in this paper did not elucidate distinctions in COVID-19 outcomes between the Arctic Indigenous populations and its non-Indigenous residents. Therefore, it is recommended that potential disparities in the impacts of COVID-19 among these populations be investigated as part of future research endeavors.

While the World Health Organization declared an end to the global Public Health Emergency for COVID-19 on May 5, 2023 (68), it is estimated that at least 65 million people experienced post-COVID-19 conditions (i.e., long COVID-19) within the initial 3 years of the pandemic (69). A multinational study by Shen et al. (70), which included 64,880 adult participants from Iceland, Sweden, Denmark, and Norway, suggested an elevated prevalence of some physical symptoms among individuals who experienced a severe acute illness, during a period extending beyond 2 years after the diagnosis of COVID-19. However, the health consequences of long COVID-19 infection at both individual and community levels in the Arctic regions are still not well comprehended. This situation presents a critical avenue for continued monitoring, shaping informed public health measures, and conducting future research.

## Data availability statement

Publicly available datasets were analyzed in this study. This data can be found here: https://univnortherniowa.maps.arcgis.com/apps/dashboards/b790e8f4d97d4414b10c03d5139ea5d5 and https://univnortherniowa.maps.arcgis.com/apps/dashboards/babca707eeac450c8604afc38230608b.

## Author contributions

ST: Conceptualization, Data curation, Formal analysis, Investigation, Methodology, Writing – original draft. AP: Conceptualization, Funding acquisition, Methodology, Project administration, Supervision, Validation, Writing – review & editing. NG: Data curation, Methodology, Visualization, Writing – review & editing. MD: Writing – review & editing. MW: Funding acquisition, Writing – review & editing. JD: Funding acquisition, Writing – review & editing. TD: Funding acquisition, Writing – review & editing. SK: Writing – review & editing.

## Footnotes

^1^ https://coronavirus.jhu.edu/map.html

^2^ https://www.folkhalsomyndigheten.se/

^3^ https://thl.fi

^4^ https://стопкоронавирус.рф

^5^ https://vg.no

^6^ https://arctic.uni.edu/arctic-covid-19
